# Effects of Pb(II) and Zn(II) Contamination on Adsorption, Desorption and Degradation of Cry1Ac Toxin Identical to Bt Transgenic Poplar in Black Soil

**DOI:** 10.3390/toxics11020089

**Published:** 2023-01-18

**Authors:** Yongji Wang, Xueyong Zhou, Fenguo Zhang, Lihong Zhang, Pingguo Yang, Rehanguli Maimaitiniyazi

**Affiliations:** School of Life Science, Shanxi Engineering Research Center of Microbial Application Technologies, Shanxi Normal University, Taiyuan 030000, China

**Keywords:** Pb(II) and Zn(II) contamination, Bt transgenic poplar, Cry1Ac toxin, adsorption, desorption, degradation

## Abstract

Bt transgenic white poplar has been commercially planted in China since 2002, and it showed obvious insect resistance in the field. However, the ecological risk of planting Bt transgenic poplar in a field contaminated with heavy metals has received little attention. The effects of Pb(II) and Zn(II) contamination on the adsorption, desorption and degradation of Bt toxin identical to Bt transgenic poplar in black soil were studied. The results showed that the adsorption of Bt toxin was enhanced and the desorption of Bt toxin was inhibited in black soil by Pb(II) and Zn(II) at concentrations between 0 and 1 mmol/L, and the effect of Pb(II) on Bt toxin was greater than that of Zn(II). In the presence of heavy metal ions, the Cry1Ac toxin molecules are oriented with domain I toward soil particles through the metal ion bridge. The promoting mechanism of Bt toxin adsorption by heavy metal ions in black soil is mainly attributed to cation-controlled electrostatic attraction (CCEA), which is different from patch-controlled electrostatic attraction (PCEA). With the increase in soil concentration from 1 to 4 mg/mL, the adsorption amount of Bt toxin showed a downward trend, and both Pb(II) and Zn(II) had the maximal promotion effect when the soil concentration was 2 mg/mL. The promoting effect of Zn(II) on the adsorption of Bt toxin increased with the increased temperature (5–45 °C), but the promoting effect of Pb(II) was maximal at 25 °C. Both Pb(II) and Zn(II) affected the degradation characteristics of Bt toxin in black soil. For the lead-contaminated black soil, the residual amount of Bt toxin increased in the early stage but decreased in the later stage compared to the control soil. For the zinc-contaminated black soil, the residual amount of Bt toxin decreased compared to the control soil except between the second and tenth days. In this study, it was observed that Bt toxin was degraded rapidly in the early stage, followed by a large amount of released Bt toxin and slow degradation in the middle and late stages.

## 1. Introduction

With the development of the wide use of forest tree products and the progressive deterioration of natural forests, extensive accelerated breeding programs are needed for reforestation and to improve existing forest tree species [1]. Genetic transformation offers an attractive alternative to breeding because it provides the potential to transfer specific traits into selected genotypes without affecting their desirable genetic background [2,3]. In this background, plant genetic transformation techniques have been used in the breeding of commercial forests. Insects can cause a large loss of forest trees, and their destruction has become a limiting factor for the growth and survival of trees [4]. In China, the poplar planting area is more than 75,700 hectares, ranking first in the world. The direct economic losses due to poplar leaf-eating pests range from USD 11.25 to 17.5 million per year [5]. In order to protect forest trees from pests, genetic engineering for insect control has been achieved in several trees using the insecticidal protein (toxin) from *Bacillus thuringiensis* (Bt) [6,7,8]. Bt transgenic white poplar has been commercially planted in China since 2002, and it showed obvious insect resistance in the field [9]. Currently, the planting area of Bt poplar has exceeded 543 hectares [10,11], and the planting area is continuing to expand. As poplar has a good tolerance to heavy metals [12], it has been used as a cultivated species in heavy-metal-contaminated areas [13,14]. Pilipović et al. [15] reported that poplar had the potential of phytoextraction and phytostabilization for soil heavy metals.

With the large-scale planting of transgenic insect-resistant poplars, their branches, leaves, falling pollen and root exudates containing Bt toxin are constantly entering the soil [16,17]. The free Bt toxin is easily adsorbed by clay minerals and humic acid from soils [18]. The binding of Bt toxin reduced its susceptibility to microbial and enzyme degradation and its insecticidal activity for more than 234 days [19]. Strain and Lydy [20] reported that the Cry1Ab protein in agricultural fields can be transported to the aquatic system and persist for up to two months. Therefore, the utilization of Bt transgenic poplar requires a comprehensive understanding of its ecological risks, especially the ecological risk of planting Bt transgenic poplars in a field contaminated with heavy metals. To our knowledge, this issue has received little attention. Previous studies investigated the effect of ionic strength on the adsorption of Bt toxin [17,21,22,23,24]; however, the influencing mechanism is not well understood. Moreover, there are inconsistencies in the earlier reports. Madliger et al. [23,24] found that Cry1Ab protein adsorption to SiO_2_ decreased with increasing *I* (ionic strength) from 10 mM to 250 mM. On the contrary, Fu et al. [25] and Zhou et al. [21] reported that the adsorption of Bt toxin increased with increasing *I* (adjusted by Na^+^, Ca^2+^ or Pb^2+^) in the low ionic strength range. The inconsistent results suggest that further studies are obviously needed to confirm the influencing characteristics and mechanism. In addition, the interaction between Bt toxin and heavy metal ions as well as the degradation characteristics of Bt toxin in heavy-metal-contaminated soil are unclear.

Currently, soil contamination with heavy metals has become a major problem that negatively affects human health and environmental safety [26]. In the past five decades, more than 800,000 tons of Pb and 30,000 tons of Cr have been released into the environment globally, most of which has accumulated in soil and thus caused serious heavy metal pollution [27]. It has been estimated that there are 3.5 million potentially contaminated sites within the European Union, of which about 0.5 million need to be remediated [28]. At present, the cultivated land polluted by heavy metals in China covers nearly 20 million hectares, accounting for about 1/6th of the total cultivated land area [29]. Therefore, the planting of Bt transgenic poplars in heavy-metal-contaminated areas may create new ecological risks. In the present study, Cry1Ac toxin, which is identical to Bt transgenic poplar, was selected as the experimental material, and the effects of Pb(II) and Zn(II) on the adsorption, desorption and degradation behavior of Cry1Ac toxin in black soil were investigated. Compared with previous studies, the innovation of this study mainly comes down to two aspects: (1) the influencing mechanism of heavy metal ions on the adsorption of Bt toxin, and (2) the degradation characteristics of Bt toxin in black soil contaminated with Pb(II) and Zn(II). This study is of great significance for evaluating the planting risk of Bt transgenic poplars in heavy-metal-contaminated soil.

## 2. Materials and Methods

### 2.1. Soil Sample

Black soil was collected from a poplar forest near the Jilin Academy of Agricultural Sciences, Gongzhuling City, Jilin Province. This plot had not been planted with Bt crops and had no heavy metal pollution. The soil sample was air-dried and passed through a 60-mesh sieve. The basic physical and chemical properties of black soil are shown in Table 1.

### 2.2. Bt Toxin and Heavy Metal Salt

The Bt toxin (Cry1Ac, which is identical to Bt poplar) was provided by the Chinese Academy of Agricultural Sciences, with a molecular weight of 60 kDa. According to the biochemical and physical properties of the Cry1 subclass toxin, the isoelectric point (IEP) of the Cry1Ac toxin is about 5.05 [30] and the hydrodynamic radius is about 5–8 nm [31,32,33]. Both Pb(NO_3_)_2_ and ZnCl_2_ were of analytical grade and purchased from Sigma Chemical Co., Ltd. (Balcatta, WA, USA).

### 2.3. Preparation of Bt Toxin Solution

Fifty milligrams of Bt toxin (Cry1Ac) powder was transferred into a triangular flask (250 mL) with 100 mL distilled water, and the pH of the suspension was adjusted to 7.0 with 0.1 mol/L sodium hydroxide. Since the dissolution of Bt toxin is slow, the Bt protein needed to continue to dissolve at 4 °C or more than 24 h. The supernatant was collected by centrifugation, and the concentration of Bt toxin was determined by using the Bt toxin standard curve.

### 2.4. Preparation of Soil Suspension

One gram of soil was dispersed in 100 mL distilled water and treated by ultrasonic bath for 30 min. The soil concentration of the suspension was 10 mg/mL.

### 2.5. Preparation of Pb (II) and Zn(II) Solution

Pb(NO_3_)_2_ and ZnCl_2_ were dissolved separately in deionized water. The concentration of the stock solution of Pb(II) or Zn(II) was 10.0 mmol/L.

### 2.6. Adsorption Tests in Batch Mode

The adsorption experiment was carried out in 50-milliliter centrifuge tubes. The total volume of the adsorption solution was 10 mL, and the initial concentration of Bt toxin and soil was 0.25 mg/mL and 1.0 mg/mL, respectively. After the Bt toxin and soil were added to the tube, the mixtures were dispersed by a vortex mixer for 30 s and then transferred into a shaking bath with a temperature of 25 ± 1 °C. The mixtures of soil and toxin in the test tubes were shaken end-over-end at 280 revolutions per minute for 2 h. After adsorption, the suspensions were centrifuged at 16,000 r/min for 20 min, and the concentration of Bt toxin in the supernatant was determined using an ultraviolet spectrophotometer at 280 nm. Since the maximum UV absorption wavelength of protein is 280 nanometers, this kind of method is more convenient than the Lowry method [34,35]. The difference between the amount of toxin added and the amount of toxin detected in the supernatant was used to calculate the amount of toxin adsorbed at equilibrium to the soil. Control experiments were performed with soil in the absence of Bt toxin.

The effect of the concentration of Pb(II) or Zn(II) on the adsorption was measured for concentrations from 0 to 1.0 mmol/L at a toxin concentration of 0.25 mg/mL and a soil content of 1.0 mg/mL. The effect of temperature on the adsorption was studied between 5 °C and 45 °C at a toxin concentration of 0.20 mg/mL and a soil content of 1 mg/mL. The effect of soil contents on the adsorption was measured for contents from 1.0 to 4.0 mg/mL and a toxin concentration of 0.20 mg/mL. The adsorption amount of Bt toxin on soil can be calculated by Equation (1).

(1) $$q_{e}=\frac{\left[C_{0}-(C_{e}-C_{w})\right]}{w}$$

where *q_e_* is the adsorption amount of Bt toxin on soil, mg/mg; C_0_ is the initial concentration of Bt toxin, mg/mL; C*_e_* is the concentration of Bt toxin adsorption equilibrium, mg/mL; C*_w_* is the background concentration of the black soil, mg/mL; and *w* is the concentration of soil, mg/mL.

### 2.7. Desorption of Bt Toxin

Bt toxin–soil complexes were prepared according to the following conditions: the initial concentration of Bt toxin was 0.2 mg/mL, the soil concentration was 1.0 mg/mL and the concentration of Pb(II) or Zn(II) was 0.05 mmol/L. After the equilibrium adsorption, the supernatant was poured out, and the water droplets on the wall of the tube were blotted with filter paper. The Bt toxin–soil complexes were washed with 4 mL of water at 25 ± 1 °C for 2 h. The concentration of Bt toxin in the supernatant was measured as described above. A control experiment was performed only with the black soil.

### 2.8. Zeta Potential of Soil Particles and Bt Toxin

The zeta potential of black soil particles and Bt toxin (a kind of colloidal solution of protein) was measured by a nanoparticle size and ZETA potential analyzer (Nano-ZS90 ZETASIZER, Malvern Instruments, Worcestershire, UK). The effects of heavy metal ions were investigated from 0 to 1.0 mmol/L.

### 2.9. Degradation of Bt Toxin in Soil

Fifty grams of black soil was added to a polyethylene plastic bag and mixed with a certain concentration of heavy metal ions. The concentration of Bt toxin in the black soil was 5.0 µg/g, and the concentration of Pb(II) or Zn(II) in the black soil ranged from 0 to 800 mg/kg. The maximum water-holding capacity was adjusted to 60% with distilled water. The soil samples were incubated in a climate chamber at 25 ± 1 °C for 0, 2, 5, 10, 18, 28 and 60 days. At each time interval, soil samples were taken out to measure the Bt toxin concentration by ELISA [36,37].

### 2.10. Statistics

All experiments were repeated four times. The data are presented as means ± standard error. Unless indicated otherwise, the standard errors of the means are within the dimensions of the figure symbols.

## 3. Results and Analysis

### 3.1. Effect of Pb(II) and Zn(II) Concentrations on the Adsorption of Bt Toxin

**Figure 1:**
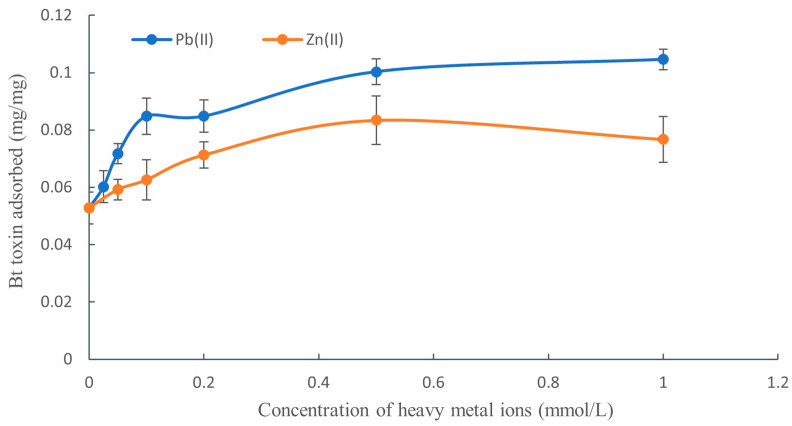
Effect of concentration of Pb(II) or Zn(II) on the adsorption of Bt toxin in black soil.

The effect of lead and zinc ions on the adsorption of Bt toxin in black soil when the concentration of Pb(II) or Zn(II) in the adsorption system ranged from 0 to 1.0 mmol/L is shown in Figure 1.

As shown in Figure 1, the adsorption of Bt toxin by black soil increased initially with the increase in the concentration of Pb(II) and Zn(II) (within 0.5 mmol/L) and then reached a more or less flat plateau. As for the promoting effect of Bt toxin adsorption, Pb(II) had a greater effect than Zn(II). Nevertheless, the concentration of Pb(II) and Zn(II) should not exceed 1.0 mmol/L, otherwise the Bt toxin begins to flocculate due to the salting-out effect.

### 3.2. Effects of Soil Concentration on the Adsorption of Bt Toxin

**Figure 2:**
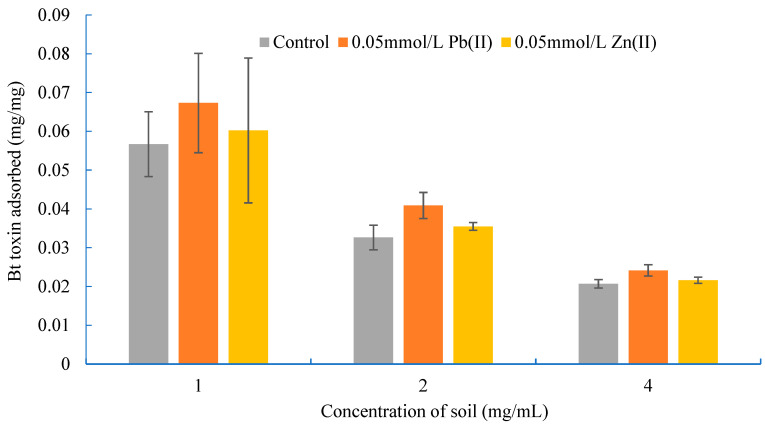
Effects of lead and zinc ions on Bt toxin adsorption under different soil concentrations.

The effects of soil concentration on the adsorption of Bt toxin when the initial concentration of Bt toxin was 0.2 mg/mL and the concentration of Pb(II) and Zn(II) was 0 and 1.0 mmol/L, respectively, are shown in Figure 2.

As shown in Figure 2, whether or not lead and zinc ions were added to the adsorption system, the amount of adsorbed Bt toxin decreased with the increase in soil concentration. However, the promoting effect of heavy metal ions on the adsorption of Bt toxin was affected by the soil concentration. For example, when the soil concentration was 1, 2 and 4 mg/mL, the promoting percentage of Bt toxin adsorption by Pb(II) (0.05 mmol/L) was 18.70%, 25.25% and 16.88%, respectively, and the promoting percentage of Bt toxin adsorption by Zn(II) (0.05 mmol/L) was 6.21%, 8.70% and 4.62%, respectively. The above results indicate that the maximal promotion effect of Pb(II) and Zn(II) occurred at a soil concentration of 2 mg/mL.

### 3.3. Effects of Temperature on the Adsorption of Bt Toxin

**Figure 3:**
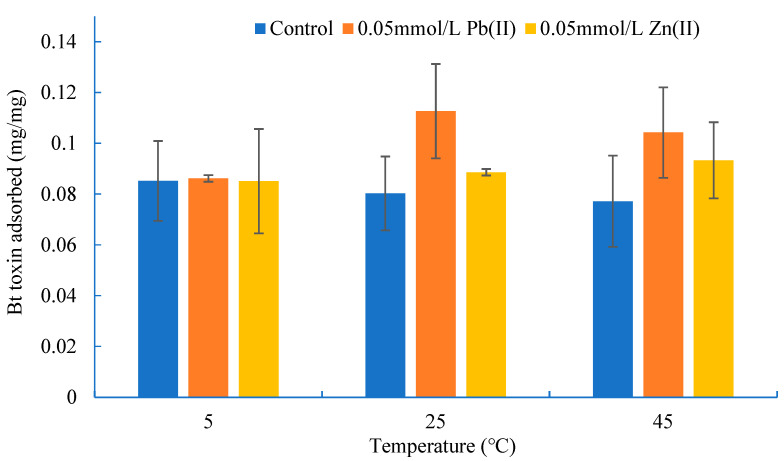
Adsorption of Bt toxin in black soil at different temperatures.

The effects of temperature on the adsorption of Bt toxin when the initial concentration of Bt toxin was 0.2 mg/mL, the soil concentration was 1.0 mg/mL and the concentration of Pb(II) or Zn(II) was 0 and 1.0 mmol/L, respectively, are shown in Figure 3.

As shown in Figure 3, the amount of adsorbed Bt toxin decreased slightly with the increase in temperature from 5 °C to 45 °C without the addition of lead and zinc ions, which agreed with previous results [38]. However, the influence trend of temperature on the adsorption of Bt toxin changed when lead or zinc ions were added to the adsorption system. For the zinc-contaminated black soil (Zn(II) was 0.05 mmol/L in the adsorption system), the adsorption of Bt toxin increased with the increase in temperature, but for the lead-contaminated black soil (Pb(II) was 0.05 mmol/L in the adsorption system), the adsorption of Bt toxin increased first and then decreased. One of the reasons for the above results may be the interaction between heavy metal ions and Bt toxin. Zhou et al. [39] reported that almost each toxin molecule binds one lead ion to form a complex. The present study showed that the promoting effect of Pb(II) (0.05 mmol/L) was higher than that of Zn(II) (0.05 mmol/L) at all temperatures.

### 3.4. The Effect of Lead and Zinc Ions on the Desorption of Bt Toxin from Black Soil

The effects of lead and zinc ions on the desorption of Bt toxin from black soil are shown in Table 2.

It can be seen from Table 2 that the desorption rate of Bt toxin from black soil without Pb(II) and Zn(II) was 32.22%; however, when Pb(II) or Zn(II) was added to the black soil, the desorption rate of Bt toxin decreased to 29.25% and 26.83%, respectively—i.e., the addition of Pb(II) or Zn(II) inhibited the desorption of Bt toxin. This is because Pb(II) and Zn(II) promoted the adsorption amount of Bt toxin in black soil (Figure 1), meaning that the affinity between the Bt toxin and the black soil particles increased, which was consistent with the results of the desorption experiment.

### 3.5. The Effect of Heavy Metal Ions on the Zeta Potential of Soil Particles

**Figure 4:**
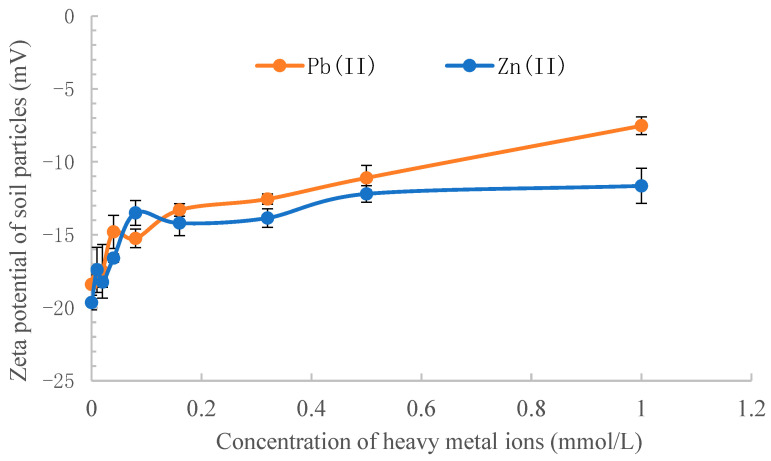
Effect of Pb(II) and Zn(II) concentration on the zeta potential of soil particles.

**Figure 5:**
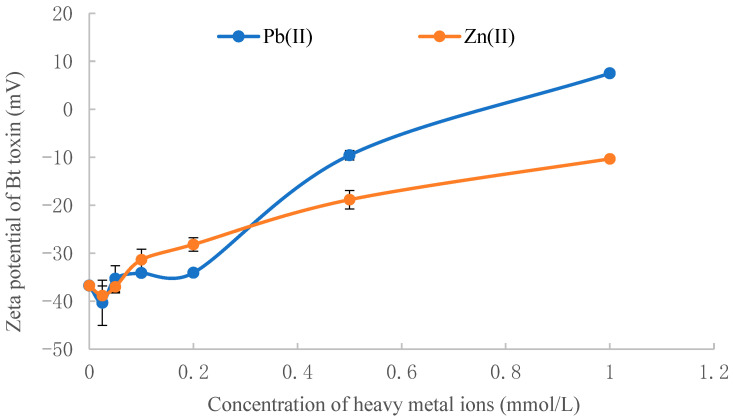
Effect of Pb(II) and Zn(II) concentration on the zeta potential of Bt toxin.

The effect of lead and zinc ions on the zeta potential of soil particles and Bt toxin is shown in Figure 4 and Figure 5. With the increase in Pb(II) or Zn(II) concentration, the zeta potential of soil particles increased rapidly at first and then gradually tended to reach equilibrium. Compared with the soil particles, the zeta potential of Bt toxin increased slowly in the initial stage; nevertheless, the changing trend was the same as the soil zeta potential (Figure 5). The effect of Pb(II) ions on the zeta potential of soil and toxin was greater than that of Zn(II) ions. As shown in Figure 4, the zeta potential of soil particles was negative before and after the addition of Pb(II) or Zn(II). The adsorption force of Bt toxin on soil mainly includes electrostatic drive, hydrophobic interaction, van der Waals force and hydrogen bonds [39]. Since the isoelectric point of Bt toxin is 5.05 [40], the surface of Bt toxin is negatively charged under the adsorption condition (pH 6.44, Table 1), which produces electrostatic repulsion with the negatively charged soil particles.

When lead and zinc ions were added to the adsorption system, the surface potential of the black soil increased, leading to a decrease in electrostatic repulsion force and an increase in adsorption capacity. The experimental results in the present study are consistent with the electrostatically driven adsorption mechanism of Bt toxin [33] but not with patch-controlled electrostatic attraction (PCEA). According to the viewpoint of Madliger et al. [23,24], PCEA results in decreasing protein affinities to sorbents of like net charges with increasing I (ionic strength) due to increasing screening of electrostatic attraction. Conversely, the affinity of a uniformly charged protein to like charged sorbents increases with increasing I (ionic strength) due to decreasing electrostatic repulsion. Apparently, our results are consistent with the latter.

**Figure 6:**
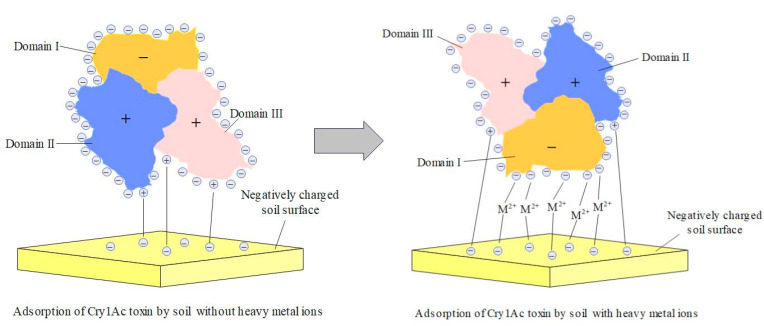
Schematic presentation for the adsorption mechanism between Cry1Ac toxin and soil particles in the presence or absence of heavy metal ions.

In fact, our results are not entirely inconsistent with those of Madliger et al. As far as the Bt toxin (Cry1Ac) is concerned, the domain I contains α-helices bearing mainly negatively charged residues, while the stacked domains II and III are mainly composed of β-sheeted structures bearing positively charged residues, such that the external protein surface has an uneven distribution of charges [17,41]. When no heavy metal ions were added to the adsorption system, the Bt toxin was adsorbed through positively charged domains II and III to negatively charged soil particles (Figure 6), i.e., the PCEA mechanism. However, when heavy metal ions were added to the adsorption system, the cations tended to interact with the negative charges on the surface of domain I to form complexes (Equation (2)):

DomainⅠ-COO^−^**+**M^2+^**→**DomainⅠ-COOM^+^ (2)

Simultaneously, the cations (heavy metal ions) also interacted with the negatively charged soil particles. Through the metal ion bridge, the Bt toxin molecules were oriented with domain I toward the soil particles (Figure 6). The above process can be regarded as cation-controlled electrostatic attraction (CCEA). It is worth noting that although the adsorption of Bt toxin through domain I predominated in the presence of heavy metal ions, the PCEA still exists. Therefore, CCEA is a complementary mechanism to PCEA.

### 3.6. Effect of Pb(II) and Zn(II) Concentration on the Degradation of Bt Toxin in Black Soil

**Figure 7:**
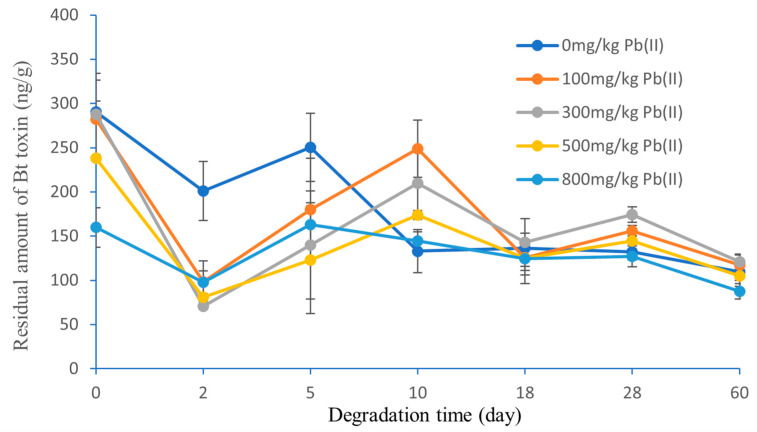
Effect of Pb(II) concentration on the degradation of Bt toxin in black soil.

**Figure 8:**
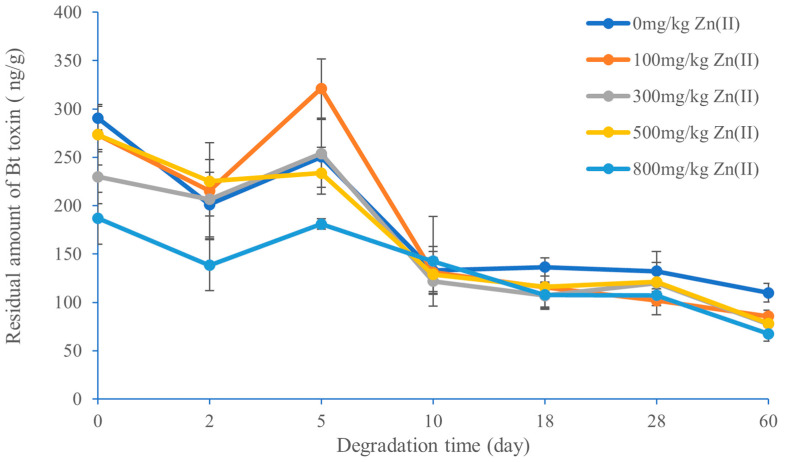
Effect of Zn(II) concentration on the degradation of Bt toxin in black soil.

The effect of Pb(II) and Zn(II) on the degradation of Bt toxin in black soil is shown in Figure 7 and Figure 8. When the Bt toxin entered the black soil (without Pb(II) or Zn(II) addition), the residual amount of Bt toxin in the black soil decreased in the first two days and then increased and reached the highest value on the fifth day. Between the fifth and tenth days, the amount of residual Bt toxin dropped rapidly again, followed by a slow decrease after the tenth day.

The degradation characteristics of Bt toxin in the lead-contaminated black soil are shown in Figure 7. The residual amount of Bt toxin in the lead-contaminated black soil decreased significantly in the first two days and then increased and reached the highest value on the tenth day. In the first five days of incubation, the residual amount of Bt toxin in the lead-contaminant black soil was lower than that in the control black soil. However, between the tenth and sixtieth days, the residual amount of Bt toxin in the lead-contaminated black soil (except for Pb(II) concentration at 800 mg/kg) was higher than that in the control black soil (Figure 7).

The degradation characteristics of Bt toxin in the zinc-contaminated black soil are shown in Figure 8. Unlike the lead-contaminated black soil, the degradation trend of Bt toxin in the zinc-contaminated black soil was similar to that in the control soil. The residual amount of Bt toxin in the zinc-contaminated black soil decreased in the first two days and then increased and reached the highest value on the fifth day. When the added concentration of Zn(II) was 800 mg/kg, the residual amount of Bt toxin was significantly lower than that in the control soil, but when the added concentration of Zn(II) was 100 mg/kg, the residual amount of Bt toxin on the fifth day was significantly higher than that in the control. Between the fifth and tenth days, the amount of remaining Bt toxin dropped rapidly again, followed by a slow decrease after the tenth day. On the whole, the residual amount of Bt toxin in the zinc-contaminated black soil (except for Zn(II) concentration at 100 mg/kg) was lower than that in the control black soil (Figure 8).

In this study, it was observed that Bt toxin was degraded rapidly in the early stage, followed by a large release of Bt toxin and a slow degradation in the middle and late stages. The degradation of Bt toxin was measured by ELISA (enzyme-linked immunosorbent assay), which was first described by Palm et al. [36]. The residual Bt toxin in soil was extracted by buffer and then quantified by the immunoreactive method. That is to say, the measured results do not necessarily equal the true residual content. Our results show that the extractable immunoreactive Bt toxin from black soil did not decrease all the time but showed one or two peaks in some stages, which is not consistent with the result of Fu et al. [22].

The release of Bt toxin in the early degradation stage was probably due to a temporary increase caused by the degradation rate being lower than the desorption rate [42]. Helassa et al. [43] observed a small release of Bt toxin at the twentieth day in soils A and B. The above results suggest that further studies are needed to accurately determine the content of Bt toxin in soils.

## 4. Conclusions

The adsorption of Bt toxin was enhanced and the desorption of Bt toxin was inhibited in black soil by Pb(II) and Zn(II) at concentrations between 0 and 1 mmol/L, and the effect of Pb(II) on Bt toxin was greater than that of Zn(II). In the presence of heavy metal ions, the Cry1Ac toxin molecules were oriented with domain I toward soil particles through the metal ion bridge. The promoting mechanism of Bt toxin adsorption by heavy metal ions in black soil is mainly attributed to cation-controlled electrostatic attraction (CCEA), which is different from patch-controlled electrostatic attraction (PCEA). The contamination of black soil by Pb(II) or Zn(II) affected the degradation trend of Bt toxin. For the lead-contaminated black soil, the residual amount of Bt toxin increased in the initial stage but decreased in the later stage. For the zinc-contaminated black soil, the residual amount of Bt toxin decreased, except between the second and tenth days. In this study, it was observed that Bt toxin was degraded rapidly in the early stage, followed by a large release of Bt toxin and a slow degradation in the middle and late stages.

## Figures and Tables

**Table 1:** Basic physical and chemical properties of black soil.

pH	AK (mg/kg)	AP (mg/kg)	AN (mg/kg)	SS (g/kg)	OM (g/kg)	Zn (mg/kg)	Pb (mg/kg)
6.44	1257.49	24.20	211.01	2.89	69.45	122.3	33.72

AK: available potassium; AP: available phosphorus; AN: available nitrogen; SS: soluble salt; OM: organic matter.

**Table 2:** Effect of lead and zinc ions on the desorption of Bt toxin in black soil.

Types of Ions	Concentration (mmol/L)	Amount of Bt Toxin Adsorbed (mg)	Amount of Bt Toxin Desorbed (mg)	Desorption Rate (%)
Pb(II)	0	0.2752	0.0887	32.22%
Pb(II)	0.05	0.3330	0.0974	29.25%
Zn(II)	0	0.2752	0.0887	32.22%
Zn(II)	0.05	0.2990	0.0802	26.83%

## Data Availability

The data presented in this article are available on request from the corresponding authors.
